# Supplementary material for: Noninvasive virtual biopsy using micro-registered optical coherence tomography (OCT) in human subjects
Source: Sci Adv. 2024 Apr 10;10(15):eadi5794. doi: 10.1126/sciadv.adi5794 (PMC11006228; doi:10.1126/sciadv.adi5794)
Supplement: 20240410-1 [file sciadv.adi5794.v1.pdf]

APPLIED SCIENCES AND ENGINEERING

Noninvasive virtual biopsy using micro-registered optical coherence tomography (OCT) in human subjects

Yonatan Winetraub[1,2,3,4]†, Aidan Van Vleck[1]†, Edwin Yuan[1,2,5]†, Itamar Terem[1,2,6]†, Jinjing Zhao[1], Caroline Yu[1,2], Warren Chan[7], Hanh Do[7], Saba Shevidi[1,2], Maiya Mao[1,2], Jacqueline Yu[1,2], Megan Hong[1,2], Erick Blankenberg[1,2], Kerri E. Rieger[8], Steven Chu[3,4,9], Sumaira Aasi[7], Kavita Y. Sarin[7], Adam de la Zerda[1,2,3,4,6,10]*

Copyright © 2024 The Authors, some rights reserved; exclusive licensee American Association for the Advancement of Science. No claim to original U.S. Government Works. Distributed under a Creative Commons Attribution License 4.0 (CC BY).

Histological hematoxylin and eosin–stained (H&E) tissue sections are used as the gold standard for pathologic detection of cancer, tumor margin detection, and disease diagnosis. Producing H&E sections, however, is invasive and time-consuming. While deep learning has shown promise in virtual staining of unstained tissue slides, true virtual biopsy requires staining of images taken from intact tissue. In this work, we developed a micron-accuracy coregistration method [micro-registered optical coherence tomography (OCT)] that can take a two-dimensional (2D) H&E slide and find the exact corresponding section in a 3D OCT image taken from the original fresh tissue. We trained a conditional generative adversarial network using the paired dataset and showed high-fidelity conversion of noninvasive OCT images to virtually stained H&E slices in both 2D and 3D. Applying these trained neural networks to in vivo OCT images should enable physicians to readily incorporate OCT imaging into their clinical practice, reducing the number of unnecessary biopsy procedures.

INTRODUCTION

Histopathology, such as hematoxylin and eosin (H&E) tissue sections, has long been the gold standard for disease diagnosis by clinicians. It provides a view of the tissue down to the micron scale and is a routine part of diagnostic procedures for both cancer (*1*) and noncancer pathologies (*2*), as well as surgical procedures such as intraoperative frozen section analysis and Mohs surgery (*3*). However, the current histopathology process is an invasive and time consuming task that can take anywhere from a few hours to several days due to its multiple processing steps, including tissue incision, formalin fixation, paraffin embedding, tissue sectioning, and staining (Fig. 1A) (*4*).

A potential alternative to histopathology is noninvasive optical imaging such as optical coherence tomography (OCT) or reflectance confocal microscopy (RCM). Studies of OCT have found high sensitivity and specificity for diagnosing certain dermatological pathologies, such as basal cell carcinoma (BCC) (*5*–*7*)—but only after the clinicians received extensive training in interpreting images. Therefore, despite that OCT and RCM's demonstrated ability to detect epithelial cancers such as skin cancer, they found only a limited clinical role as a diagnostic tool for these cancers in practice (*8*, *9*). A tool that converts noninvasive OCT images to an H&E-like format using machine learning may improve the diagnostic power of the clinician without the need for a biopsy (Fig. 1B).

[1]Department of Structural Biology, Stanford University, Stanford, CA 94305, USA. [2]Molecular Imaging Program at Stanford, Stanford, CA 94305, USA. [3]The Bio-X Program, Stanford, CA 94305, USA. [4]Biophysics Program at Stanford, Stanford, CA 94305, USA. [5]Department of Applied Physics, Stanford University, Stanford, CA 94305, USA. [6]Department of Electrical Engineering, Stanford University, Stanford, CA 94305, USA. [7]Department of Dermatology, Stanford University School of Medicine, Stanford, CA 94305, USA. [8]Department of Pathology, Stanford University School of Medicine and Stanford Cancer Institute, Stanford, CA 94305, USA. [9]Departments of Physics and Molecular and Cellular Physiology, Energy, Science and Engineering Stanford University, Stanford, CA 94305, USA. [10]The Chan Zuckerberg Biohub, San Francisco, CA 94158, USA.
*Corresponding author. Email: adlz@stanford.edu
†These authors contributed equally to this work.

In recent years, deep learning–enabled virtual histology has shown promise in virtual staining of unstained slides (*10*–*13*). However, a key challenge remains—how to virtually stain images captured noninvasively. Most machine learning image-to-image translation models are being trained on paired image sets, imaging the exact same area using both modalities. A key component of paired image sets is the need to precisely coregister images obtained from both modalities.

Coregistration is relatively simple when the two modalities are slide images as the on-slide image deformation during staining is usually local and minimal. Coregistration becomes much more difficult, however, when registering fresh tissue images with slides due to the increased degrees of freedom. Although it is possible to avoid the registration problem by staining the bulk tissue (*14*) and imaging H&E-like slides without obtaining histological sections, this approach is prone to imaging artifacts as image depth increases.

To enable virtual histology based on OCT imaging, each OCT image in the training set must be registered to its corresponding H&E image with the highest precision possible (*15*). High-resolution registration of optical images with H&E images requires a precise match in nine degrees of freedom (three translations, three axis rotations, and shrinkage/scale change in all axes), which had not previously been adequately achieved (*16*), and certainly not in a systematic and repeatable manner (*16*). Previous work relied on tissue landmarks, careful orientation of tissue cutting, and even luck to arrive at an acceptable OCT-H&E image pair (*16*, *17*). Even in the best case scenario, it was difficult to correlate tissue features in the OCT with those in the H&E image with a precision of less than 500 μm at best. Virtual H&E staining from noninvasive optical images requires substantial improvement in the coregistration precision.

RESULTS

Coregistration

To precisely coregister OCT and histology slides, we developed a method, micro-registered OCT, that allowed us to register the

two-dimensional (2D) plane within a 3D OCT volume while finding the corresponding 2D histology image (Fig. 2). We encased freshly excised tissue samples within a transparent fluorescent agar-gelatin gel (Fig. 2; see also Materials and Methods below and the Supplementary Materials). We then acquired a 3D OCT volume image of the tissue within the gel and lastly photobleached a specific geometric pattern (barcode) into the gel by a second laser wavelength sent through the OCT optical system (Fig. 2A). This pattern forms a barcode marker that is preserved through the standard histological process. Our coregistration algorithm assumes a histological section is approximated by a 2D plane and uses the markers to estimate the nine parameters that define how the plane was cut: plane orientation, position, and scaling (Fig. 2B). Plane parameters are averaged across consecutive histological sections to reduce our estimator's noise. We performed additional manual plane parameter refinement based on landmarks found on the tissue such as skin surface shape.

To validate coregistration quality, we applied our micro-registration method to align histology sections from several human skin samples freshly excised during Mohs surgery (IRB-24307). We embedded 25-μm Poly(lactic-co-glycolic acid) fluorescent beads to the gel and were able to visualize the same beads in both the 2D OCT plane that was estimated by the registration algorithm and the histological section (fig. S7). Because the beads were scattered throughout the section and their position matched between histology and OCT plane, we believe that the plane was predicted correctly to the accuracy of one bead width or about 25 μm. In addition, we attempted to directly estimate the 2D OCT plane using the beads alone without using the barcode pattern and were unsuccessful to find a match. The number of possible "hits" was large enough, such that it was unclear which 2D plane was the one that represented the section the most reliably.

In another validation effort, we selected 11 human skin samples (82 sections) in which OCT image quality was high and histology sections presented distinctive small features such as small hair follicles or epidermis-dermis junction's shape. These features should also be visible in OCT and thus can be used to validate the registration. Examples of these image pairs can be found in figs. S8, S10, and S11. We manually validated the quality of the registration by examining nearby 2D planes paralleling the coregistered OCT 2D plane. For example, fig. S11 shows a hair follicle visible in histology (fig. S11A) and in the coregistered OCT 2D plane (fig. S11B). When examining nearby parallel 2D planes 25 μm away from the plane in fig. S11B, we see that the hair follicle is no longer visible in OCT (fig. S11, C and D), providing an upper bound on cross 2D plane registration accuracy. When repeating this process for 82 samples, we see that the registration accuracy is 59 μm (SD) for the initial 2D plane estimation (before the manual refinement step described above) and about 25 μm (SD) after the manual refinement step, more than an order of magnitude better than the state of the art.

We believe that cross-plane alignment accuracy was the main contributor to coregistration error because any in-plane errors are easily addressed by manually stretching or panning the OCT image until it fits the histology image as can be done in the manual refinement step above. We can bound the orientation errors by examining small features that are present throughout the OCT and histology images. For example, in fig. S10, in order for the epidermis-dermis junction to remain visible through the entire OCT 2D plane, orientation error must be smaller than 25 μm over the length of the scan—about 1 mm—or orientation error of a few degrees at most. We can further validate orientation error by looking at the cross-plane error of consecutive sections in the same OCT volume. For example, fig. S20 shows multiple consecutive OCT 2D planes that have landmarks showing good registration with histology sections.

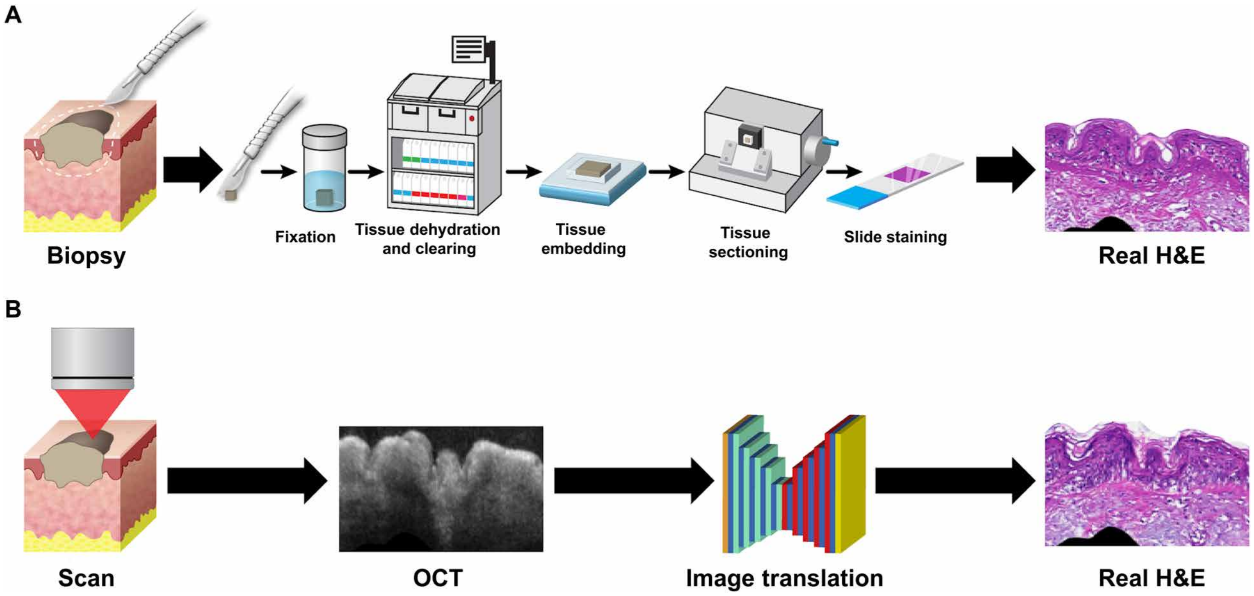

Fig. 1. Traditional biopsy versus virtual biopsy. (**A**) In a traditional biopsy, tissue is first excised and then undergoes multiple steps including fixation, dehydration, clearing, embedding, sectioning, and staining to yield 2D H&E sections, which can be examined under a microscope. (**B**) For a virtual biopsy, an OCT scan of tissue is acquired, and a trained neural network transforms the 2D OCT image into a corresponding H&E-like image.

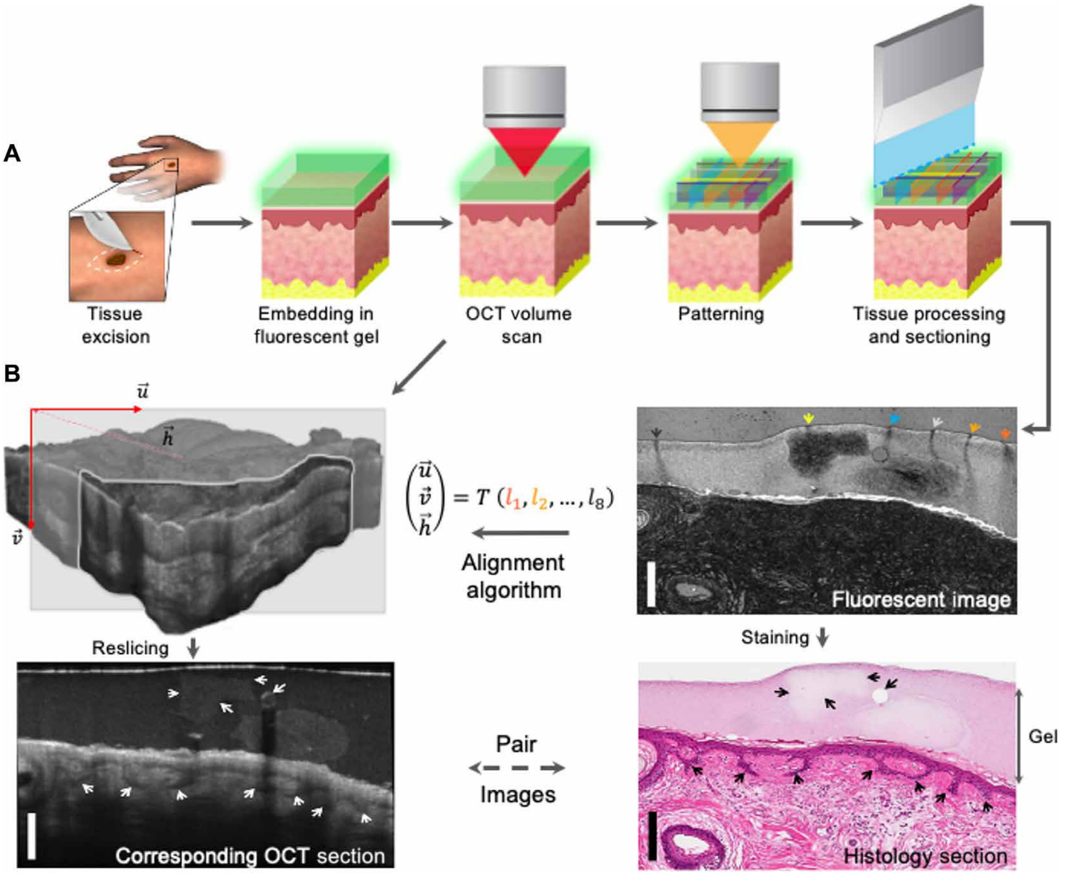

Fig. 2. Illustration of the micro-registration method used to create precisely aligned OCT-H&E image pairs from fresh tissue samples. (**A**) Top row (left to right): The collected tissue specimen is encased in the fluorescent gel. After an OCT volume scan is taken and a diode laser patterns a barcode by photobleaching the fluorescent gel, the tissue undergoes histological processing and tissue sectioning. Note that the colored lines in (A) are in reality dark photobleached lines, coloring is for illustration only. (**B**) Tissue section is imaged (top right) to produce a 2D fluorescence image containing the barcode pattern (denoted by the colored arrows) and then H&E stain (bottom right). The barcode is used to reslice the 3D OCT volume (top left) and to extract a 2D OCT image (bottom left) that physically corresponds to the H&E section (bottom right). The black and white arrows pointing to strands of epithelial cells in the epithelium and a gelatin chunk in the tissue-encasing gel in both H&E and its corresponding OCT images. Scale bars, 300 μm.

We therefore conclude that orientation errors are bounded by the cross-plane error.

We further examined whether micro-registered OCT could be reproduced using different objective lenses. The tests mentioned above were using a ×10 lens; however, some conditions, such as melanoma, require a ×40 lens. In fig. S20, we demonstrate precise coregistration of OCT and histology slides on ×40 level as well. The higher magnification OCT images contain cellular information and could be used to generate an OCT to histology model that reliably reproduces cellular features in the future.

OCT to histology model

Next, we created a paired image dataset for machine learning training. Of 119 human skin samples freshly excised during Mohs surgery (IRB-24307), we removed samples with low-quality registration or low quality of either OCT or H&E images and arrived at a dataset of 1005 coregistered OCT-H&E image pairs (one image pair per histology section) taken from 71 of the skin samples. The dataset was subject to further digital processing for stain color normalization and removal of low signal-to-noise ratio (SNR) image regions before the images were used for the machine learning task. These image pairs were coregistered with sufficient precision to train a conditional generative adversarial network (cGAN) to perform image-to-image translation from OCT images to H&E-like images.

To produce our image translator, we developed a conditional GAN model based on the pix2pix framework, but with some changes that were found to improve the quality of the generated images. We trained our cGAN model (*15*) on the dataset of 553 high-quality OCT-H&E image pairs from 38 patients (54% of dataset). We then tested its performance on patient samples that it had not encountered before (452 high-quality OCT-H&E image pairs from the other 33 patients). Our training data included a large number of skin samples from the scalp and regions of the face and neck and smaller numbers from the legs and chest (Fig. 3A and figs. S14 and S15).

Figure 3 (B to D) shows example test results of OCT images from the face and forearm, each one alongside the virtual H&E generated by the neural network, and the real H&E that had been coregistered with the OCT slice by our micro-registered OCT method. In the OCT images, the nuclei-rich epithelial layer appears darker, while

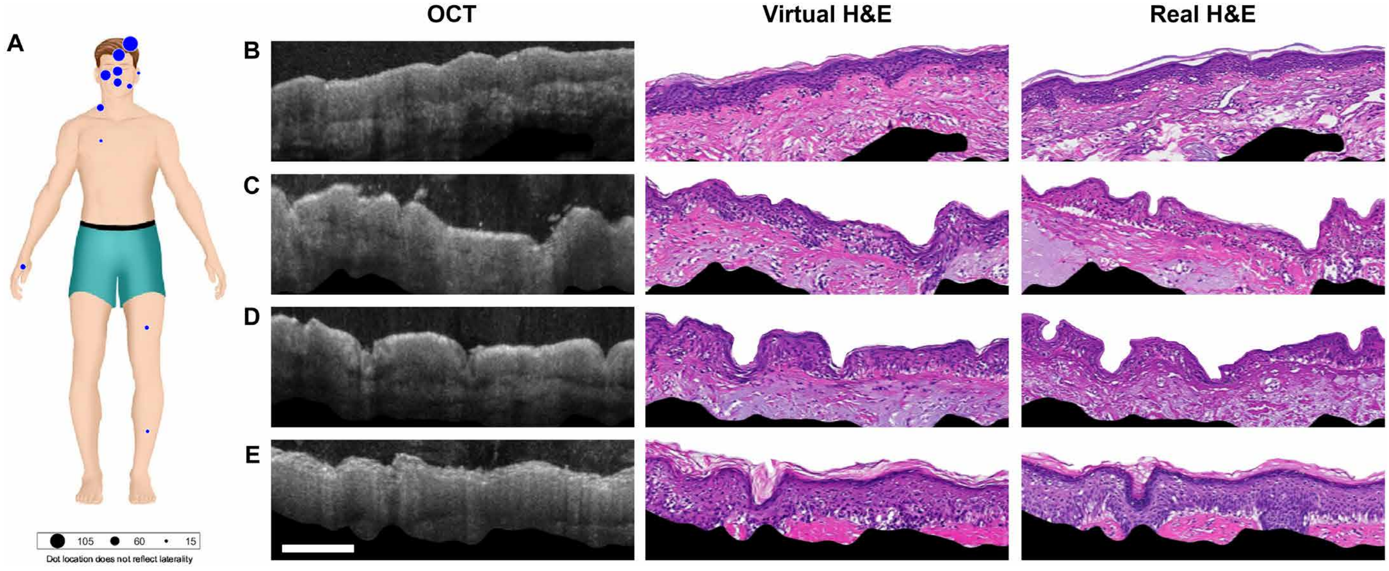

Fig. 3. Generating virtual histology H&E images from OCT. (**A**) Locations of biopsies used in our training set (blue dots). Panel at right shows examples (from the testing set) of an OCT image, the computer generated virtual H&E image, and the corresponding actual histology image from face region where we had a dense training set (**B** to **D**) and from the forearm (**E**) where we had no nearby training set. Features as small as tens of microns in size, as found in the shape of the epithelium, are matched. We do not expect smaller features to match as our OCT scanner does not have the resolution or information density to accurately generate smaller features. Scale bar, 200 μm.

the highly scattering connective tissue of the stroma tends to appear brighter. The generated H&E images demonstrate the neural network's understanding of these features.

The first sample from the face (Fig. 3B) has a well-defined epithelial layer and strong signal and contrast within the stroma. Notice that the epithelium shrinks during histological processing, and the image translator takes this into account, accurately reproducing the epithelial thickness of the H&E images. Part of a hair follicle is also visible on the left side of the H&E image. The follicle is less apparent in the virtual H&E image, possibly due to imperfect registration or difficulty seeing it in the OCT image. The final sample (Fig. 3D) is of arm skin with ridge-like epithelium. The neural network generated a high-quality H&E virtual image despite seeing no examples of arm skin in its training set. Note that some features on the OCT image do not translate perfectly to the real H&E images, likely due to imperfections of the alignment algorithm.

To assess the ability of the virtual histology algorithm to clinically replicate normal skin structures and to quantify its potential clinical utility, we examined how accurately the virtual histology reproduces hair follicle structures. Dermatopathologists count and identify hair follicles in histological images to evaluate for alopecia (hair loss) conditions, highlighting a potential clinical application of our technology. We selected 80 images from the test, maximizing diversity of tissue types, location, and subjects. Forty of those images were ground truth histology images, and 40 were the corresponding virtual histology images. We estimate the physical distance between the virtual histology and ground truth histology to be around 25 μm. Three dermatologists (including one dermatopathologist) randomly shuffled and then evaluated the images for the presence of hair follicles in each image (one at a time). The evaluation was done in a double-blind manner, presenting one image at a time. When coders did not agree, we used majority rule to determine the image diagnosis. Of the 40 image pairs, 22 were reported with hair follicle and 18 without, returning a sensitivity of 85% and a specificity of 100%. We observed four cases of false positives (hair follicle is absent according to ground truth but is present according to virtual histology) and no false negatives (hair follicle is present according to ground truth but is absent according to virtual histology). We believe that by further increasing the training set, we can improve neural network performances even further (see the Supplementary Materials).

Notably, our virtual histology has the potential to provide information beyond that achieved with traditional histology sections. By generating virtual H&E images slice by slice, we can also create an entire virtual H&E volume from a corresponding OCT volume (Fig. 4). The virtual H&E volume can be resliced from either axis (Fig. 4, B and C, and see also movie S1). The ability to create such a representation provides a powerful, efficient and intuitive way of collecting H&E-like data from a patient and presenting it to a clinician, avoiding the randomness and imprecision of the sectioning process (*18*, *19*), where only a few slices from an entire tissue block can be examined under a microscope.

DISCUSSION

Our micro-registration method has shown the ability to align fresh tissue OCT scans with histological slides to an accuracy of 25 μm (59 μm if manual refinement step is excluded), more than an order of magnitude better than the state of the art. Our method is able to perform micro-registration at a high yield on >60% of samples. Furthermore, we believe that our method is applicable to many noninvasive optical imaging tools (e.g., confocal reflectance microscopy, intravital microscopy, and photoacoustic imaging) and can work in multiple magnification lenses, enabling any of these imaging tools to

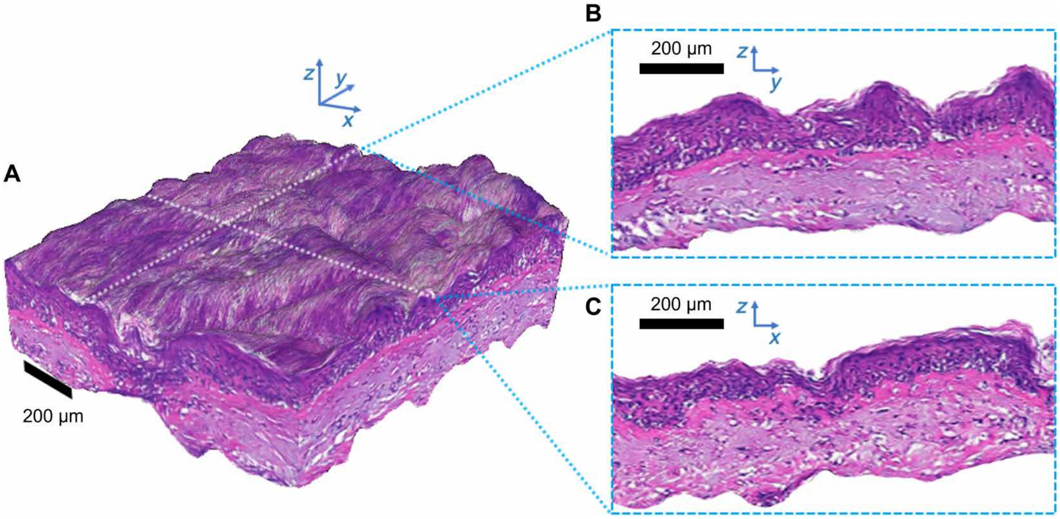

Fig. 4. A 3D virtual H&E volume generated slice by slice from an OCT volume. The volume can be viewed in multiple ways, including isometric projection (**A**) and 2D cross-sectional views from within the volume (**B** and **C**), which can be chosen to slice across any plane.

perform virtual biopsies powered by machine learning at a cellular resolution.

Our work has demonstrated a proof of concept of the 3D virtual biopsy approach: the generation of highly realistic virtual H&E sections directly from OCT images. This result in turn relied on the feasibility of using our micro-registration method to create a large database of accurately coregistered pairs of OCT and H&E histology images. These aligned image pairs were essential for generating high quality virtual histology images with our cGAN.

We note that recent works (*20*–*22*) predict histology stains from unstained slides using unpaired image sets that are presented as not requiring coregistration. That said, although the image set is unpaired, the unstained and stained slides are mostly coregistered because they are images of the same slide. We performed tests using an alternative cGAN that is trainable with unregistered and unpaired images, which generated output images of much lower quality (fig. S13). We therefore assume that our coregistration method is beneficial for unpaired image sets that are needed to train a model that converts fresh tissue to H&E slides.

There are two primary directions for taking this work further, which we anticipate will open the door for a variety of basic research and clinical applications. First, our micro-registration method can be used to obtain the precise H&E ground truth of OCT images and other types of optical image. Obtaining reliable ground truth enables multiple applications, such as verifying the binding of contrast agents, better understanding how different structures may be distorted in histology, or for obtaining data needed to train other machine learning algorithms, such as those designed to directly classify cancers or other abnormalities from an OCT image.

Second, our method can be applied and further developed for a variety of basic and clinical research applications. Once it is properly trained, the image translator can be used to produce virtual H&E images without any need to micro-register the OCT-imaged tissue. Thus, it can be used noninvasively in vivo either to diagnose various superficial skin cancers or to confirm tumor margins, bypassing the invasive, time-consuming, and costly biopsy and histological processing procedure (*4*). This is particularly important in cases where small tumors may have been completely eradicated by the biopsy and subsequent cascade of inflammation. In these cases, no noninvasive modality is available to determine whether surgery is still indicated without making an incision that can lead to scarring on the patient's skin, which is particularly undesirable on the face (*23*).

We note several shortcomings of the present machine learning algorithm. The first is that because we had to run on freshly excited tissues, we used a relatively small dataset to train our model of 1005 images from 71 patients. Modern cGANs are trained on an order of magnitude more data, which makes the model more robust and generalizable to not previously seen samples. More specifically, we acknowledge that thick stratum corneum is not well reproduced due to the limited representation of thick stratum corneum in the training set. In addition, our training dataset is not balanced across perceived Fitzpatrick skin type. We expect our model to generate even more robust epithelial structures, textures, and stromal features as the training dataset increases and diversifies. As is well documented for deep learning applications, we expect the quality and robustness of our results to scale with the amount of data collected (*24*, *25*).

A second limitation is the relatively small amount of information present in OCT images due to resolution, signal quality, and contrast mechanism. OCT image resolution sets a lower limit on the size of features that can be reliably generated by the neural network. In practice, speckle noise (*26*–*28*) and multiple scattering (*29*) can further obfuscate the useful information within a voxel. For this reason, in this paper we focused on generating large features (>50 μm; see fig. S22 for quantification) such as tissue layers and hair follicles that have sufficient reliable information in the OCT image. Although our algorithm generates fine histology features, such as cell nuclei, we believe that they should be regarded as general texture features and not interpreted as the exact position of individual cells, as current OCT signal quality does not allow us to capture this information. In our training and testing, we specifically addressed the issue of lack of OCT signal in certain regions of the image (due to optical extinction) by masking out low-signal regions in both OCT and H&E images. The optical extinction also limits the imaging

depth, which we typically observed was up to 450 μm for reasonable SNR for the machine learning algorithm. We believe that future work based on OCT systems with higher spatial resolution, such as commercial systems with the capability to reliably image single cells (*30*–*32*), will yield virtual H&E images of substantially higher quality. The principles developed in this work are easily translatable to these higher resolution OCT systems.

We believe that our method can pave the way for a true virtual biopsy that is able to generate intuitive H&E-like images, supporting clinical decision-making in dermatology by identifying tumor margins and confirming tumor diagnosis. We further believe our micro-registration method can shed light on the relationship between structures seen in OCT, confocal microscopy, and other optical methods and a histopathological section of the exact same area.

An immediate next step will be to demonstrate that the model can robustly generate H&E-like features of superficial BCC, including cancer margins, from OCT images. This is a favorable test case, as BCC clusters are typically hundreds of microns in size (*9*, *33*), putting them well within the range of our system. As a preliminary result, we added a few cancer samples to our training set and showed feasibility for noninvasive BCC detection (fig. S18).

MATERIALS AND METHODS

Experimental design

Our micro-registration and cGAN-training pipeline featured several steps: encasing the tissue in fluorescent gel, OCT imaging, writing the barcode pattern by photobleaching, computationally extracting the reslice parameters from the barcode, user-performed fine alignment of the OCT-H&E image pairs, quality assurance, stain color normalization, removal of low-SNR regions from image, and lastly machine learning training. Additional information is in the Supplementary Materials.

Sample extraction

All participants provided verbal consent to participate in this study under IRB-24307. We received fresh tissue samples daily from Mohs surgery operations. Samples were stored and transferred in keratinocyte media consisting of Dulbecco's modified Eagle medium/F12 containing penicillin/streptomycin (0.1%), fungizone (40 μg/ml), B27 (without vitamin A), epidermal growth factor (20 ng/ml), and basic fibroblast growth factor (40 ng/ml; Sigma-Aldrich) to support cell viability and were processed within 4 hours after excision. Before processing, tissue samples were transected to create a smaller size of approximately 0.5 cm by 0.25 cm by 0.5 cm.

Gel encasement

Each tissue block was encased in a fluorescent gel. The gel was produced by mixing 0.03 g of Knox gelatin with water and 50 μl of 50 μM Alexa 680–*N*-hydroxysuccinimide Ester dye in a glass flask and mixing on a tilt table for 10 min. We then added 0.06 g of agar and an additional 0.045 g of gelatin to the mixture before bringing the mixture to a boil in the microwave (about 10 s). The mixture was cooled for 10 s and then poured over the tissue sample resting in a cassette. After about 5 min at room temperature, the liquid mixture completely solidified to a gel.

OCT imaging and barcoding

Each encased sample was placed on an XYZ translation stage (Thorlabs MT3-Z8) for imaging and barcoding. All images were collected with a Thorlabs Ganymede OCT system (Thorlabs GAN220). The optical fiber transmitting light to the scanhead was replaced with a custom-made wavelength division multiplexer with two input ports (for the photobleaching laser and the OCT source) and one output port. The OCT system was outfitted with a 10×, long working distance objective lens (Olympus UMPLFLN10XW) with an effective numerical aperture of 0.2, which was used with silicone oil immersion (n = 1.4, which approximately matches the refractive index of skin tissue). Theoretical lateral resolution of the system is 3.7 μm, and the axial resolution is 2 μm.

We collected 48 volume scans of 1 mm by 1 mm, where the optical focus was translated by 10 μm between each scan by a z-axis translation of the sample. All volumes were then computationally stitched together to yield an OCT volume with isotropic lateral resolution. After imaging was completed, a 650-nm laser diode (LP660-SF50) was activated (about 3.3 mW at output) to photobleach the barcode pattern with two passes per line, at a line rate of 1 mm/15 s. Afterward, we took an overview scan (fig. S4) of total size of 7 mm by 8 mm, consisting of tile scans of 1 mm by 1 mm. The overview scan was used to determine a specific distance to cut into the tissue block to reach the location of the barcode pattern.

H&E preparation

The gel-encased, bar-coded tissue was then submerged in formalin solution for 24 hours and then transferred to 30% ethanol solution for about 2 hours. The sample then underwent histological processing (protocol is shown in fig. S5) and the two-iteration cutting procedure (described in the "Histological processing and tissue sectioning" section in the Supplementary Materials), which was designed to capture tissue sections in the most optimal location relative to the barcode pattern. From each tissue sample, we ultimately received 15 unstained tissue sections, which were imaged in a Leica SP5 microscope with a 633-nm emission filter to capture the fluorescent barcode pattern on each section. The sections were then H&E stained and white light images scanned at ×20 magnification in an Aperio Digital Pathology Slide Scanner.

Registration

The H&E sections were aligned to resliced OCT images using in-house code that allows the user to select the photobleached lines from each fluorescent slide image. The registration parameters computed directly from the algorithm constitute the "stack alignment." We then performed a manual "fine alignment" step, where the resliced OCT image was fine-tuned to match the H&E image by adjusting the translation in three axes and in-plane rotation.

Images were filtered based on H&E image quality, OCT image quality, and registration quality. We cropped OCT-H&E image pairs to 1024 × 512 pixels at ×10 magnification (1 μm/pixel). We masked out areas of the image where the OCT signal did not have a sufficient SNR to prevent the neural network from hallucinating tissue features where there is low signal in the OCT image. The results in this work were obtained by training the pix2pix model on the OCT-H&E image crops, which are automatically resized to 256 × 256 at runtime within the pix2pix preprocessing code. The model also outputs test results at 256 × 256, which are resized within an external code base to 1024 × 512.

The pix2pix model was modified to use the resnet9 generator, with 50% dropout and with additional training-time data augmentation of random image translation. Use of the resnet9 generator

(11.4 million parameters), compared to the baseline U-net network (54.4 million parameters), was found to improve generator mode collapse issues. The random translations, which consisted of random translations of up to 50% of the image's length on each axis, were found to improve the quality of generated image features, such as hair follicles and epithelial structures.

Statistical analysis

To verify the coregistration accuracy between OCT scans of freshly excised tissue and H&E slides, we used two independent methods (see the Supplementary Materials for additional information). We first placed fluorescent beads of 25-μm diameter int encased in a gelatin gel. The beads are visible in OCT, fluorescence confocal microscopy, and in histological sections after the full histological processing. In figs. S6 and S7, we can verify the coregistration accuracy by the distance between the beads in both modalities. We have successfully reproduced this bead experiment multiple times, and thus, this is one method through which we have concluded that our alignment algorithm is capable of alignment precision down to 25 μm. We can also observe the success of the optical barcoding technique by examining aligned OCT-H&E image pairs and comparing the location of identifiable landmarks in both modalities (see examples in figs. S8 and S10). We repeated the process for 82 sections (fig. S9) to compute the average coregistration accuracy.

We trained our cGAN model on the dataset of 553 high-quality OCT-H&E image pairs from 38 patients (54% of dataset). We then tested its performance on patient samples that it had not encountered before (452 high-quality OCT-H&E image pairs from the other 33 patients). To assess the ability of the virtual histology algorithm to clinically replicate normal skin structures and to quantify its potential clinical utility, we examined how accurately the virtual histology reproduces hair follicle structures. Dermatopathologists count and identify hair follicles in histological images to evaluate for alopecia (hair loss) conditions, highlighting a potential clinical application of our technology. We selected 80 images from the test, maximizing diversity of tissue types, location, and subjects. Forty of those images were ground truth histology images, and 40 were the corresponding virtual histology images.

Supplementary Materials

This PDF file includes:
Supplementary Text
Figs. S1 to S23
Legend for movie S1
References

Other Supplementary Material for this manuscript includes the following:
Movie S1

REFERENCES AND NOTES

1. F. C. Eberle, H. Mani, E. S. Jaffe, Histopathology of Hodgkin's lymphoma. *Cancer J. Sci. Am.* **15**, 129–137 (2009).
2. E. Gupta, P. Bhalla, N. Khurana, T. Singh, Histopathology for the diagnosis of infectious diseases. *Indian J. Med. Microbiol.* **27**, 100–106 (2009).
3. K. Mariwalla, S. Z. Aasi, E. J. Glusac, D. J. Leffell, Mohs micrographic surgery histopathology concordance. *J. Am. Acad. Dermatol.* **60**, 94–98 (2009).
4. D. S. Strayer, J. E. Saffitz, E. Rubin, *Rubin's Pathology: Mechanisms of Human Disease* (Lippincott Williams & Wilkins, 2019).
5. J. Fisher, D. M. Siegel, J. Fisher, O. Markowitz, D. M. Siegel, O. Markowitz, Clinical utility of bedside multibeam optical coherence tomography imaging in a patient with multiple basal cell carcinomas. *Dermatol. Surg.* **44**, 874–876 (2018).
6. M. Ulrich, T. von Braunmuehl, H. Kurzen, T. Dirschka, C. Kellner, E. Sattler, C. Berking, J. Welzel, U. Reinhold, The sensitivity and specificity of optical coherence tomography for the assisted diagnosis of nonpigmented basal cell carcinoma: an observational study. *Br. J. Dermatol.* **173**, 428–435 (2015).
7. G. Li, J. K. Tietze, X. Tao, D. Kulichova, T. Ruzicka, C. Berking, T. Maier, High-definition optical coherence tomography in the diagnosis of basal cell carcinoma evaluated by an experienced versus inexperienced investigator. *J. Clin. Exp. Dermatol. Res.* **5**, 4 (2014).
8. D. Cunha, T. Richardson, N. Sheth, G. Orchard, A. Coleman, R. Mallipeddi, Comparison of ex vivo optical coherence tomography with conventional frozen-section histology for visualizing basal cell carcinoma during Mohs micrographic surgery. *Br. J. Dermatol.* **165**, 576–580 (2011).
9. M. A. L. M. Boone, S. Norrenberg, G. B. E. Jemec, V. Del Marmol, Imaging of basal cell carcinoma by high-definition optical coherence tomography: Histomorphological correlation. A pilot study. *Br. J. Dermatol.* **167**, 856–864 (2012).
10. N. Pillar, A. Ozcan, Virtual tissue staining in pathology using machine learning. *Expert Rev. Mol. Diagn.* **22**, 987–989 (2022).
11. K. de Haan, Y. Zhang, Y. Rivenson, J. Li, A. Delis, A. Ozcan, Multiplexed and micro-structured virtual staining of unlabeled tissue using deep learning, in *Emerging Topics in Artificial Intelligence* (SPIE, 2020).
12. R. Cao, S. D. Nelson, S. Davis, Y. Liang, Y. Luo, Y. Zhang, B. Crawford, L. V. Wang, Label-free intraoperative histology of bone tissue via deep-learning-assisted ultraviolet photoacoustic microscopy. *Nat. Biomed. Eng.* **7**, 124–134 (2023).
13. E. M. Christiansen, S. J. Yang, D. M. Ando, A. Javaherian, G. Skibinski, S. Lipnick, E. Mount, A. O'Neil, K. Shah, A. K. Lee, P. Goyal, W. Fedus, R. Poplin, A. Esteva, M. Berndl, L. L. Rubin, P. Nelson, S. Finkbeiner, In silico labeling: Predicting fluorescent labels in unlabeled images. *Cell* **173**, 792–803.e19 (2018).
14. J. Li, J. Garfinkel, X. Zhang, D. Wu, Y. Zhang, K. de Haan, H. Wang, T. Liu, B. Bai, Y. Rivenson, G. Rubinstein, P. O. Scumpia, A. Ozcan, Biopsy-free in vivo virtual histology of skin using deep learning. *Light Sci. Appl.* **10**, 233 (2021).
15. P. Isola, J.-Y. Zhu, T. Zhou, A. A. Efros, Image-to-image translation with conditional adversarial networks, in *2017 IEEE Conference on Computer Vision and Pattern Recognition (CVPR)* (IEEE, 2017).
16. N. Chicherova, K. Fundana, B. Müller, P. C. Cattin, Histology to microCT Data matching using landmarks and a density biased RANSAC. *Med. Image Comput. Comput. Assist. Interv.* **17**, 243–250 (2014).
17. D. Yecies, O. Liba, E. D. SoRelle, R. Dutta, E. Yuan, H. Vogel, G. A. Grant, A. de la Zerda, Speckle modulation enables high-resolution wide-field human brain tumor margin detection and in vivo murine neuroimaging. *Sci. Rep.* **9**, 10388 (2019).
18. K. K. Mehra, M. C. Chang, A. K. Folkins, C. J. Raho, J. F. Lima, L. Yuan, M. Mehrad, S. S. Tworoger, C. P. Crum, A. Saleemuddin, The impact of tissue block sampling on the detection of p53 signatures in fallopian tubes from women with BRCA 1 or 2 mutations (BRCA+) and controls. *Mod. Pathol.* **24**, 152–156 (2011).
19. S. W. Dyson, J. Bass, J. Pomeranz, C. Jaworsky, J. Sigel, S. Somach, Impact of thorough block sampling in the histologic evaluation of melanomas. *Arch. Dermatol.* **141**, 734–736 (2005).
20. L. Kang, X. Li, Y. Zhang, T. T. W. Wong, Deep learning enables ultraviolet photoacoustic microscopy based histological imaging with near real-time virtual staining. *Photoacoustics* **25**, 100308 (2022).
21. Y. Lin, B. Zeng, Y. Wang, Y. Chen, Z. Fang, J. Zhang, X. Ji, H. Wang, Y. Zhang, Unpaired multi-domain stain transfer for kidney histopathological images. *AAAI Conf. Artif. Intell.* **36**, 1630–1637 (2022).
22. N. Bouteldja, B. M. Klinkhammer, T. Schlaich, P. Boor, D. Merhof, Improving unsupervised stain-to-stain translation using self-supervision and meta-learning. *J. Pathol. Inform.* **13**, 100107 (2022).
23. P. L. Bailin, M. D. Bailin, Correction of depressed scars following Mohs' surgery: The role of collagen implantation. *J. Dermatol. Surg. Oncol.* **8**, 845–849 (1982).
24. J. Cho, K. Lee, E. Shin, G. Choy, S. Do, How much data is needed to train a medical image deep learning system to achieve necessary high accuracy? arXiv:1511.06348 [cs.LG] (19 November 2015).
25. G. Chartrand, P. M. Cheng, E. Vorontsov, M. Drozdzal, S. Turcotte, C. J. Pal, S. Kadoury, A. Tang, Deep learning: A primer for radiologists. *Radiographics* **37**, 2113–2131 (2017).
26. J. M. Schmitt, S. H. Xiang, K. M. Yung, Speckle in optical coherence tomography. *J. Biomed. Opt.* **4**, 95 (1999).
27. Y. Winetraub, C. Wu, G. P. Collins, S. Chu, A. de la Zerda, Upper limit for angular compounding speckle reduction. *Appl. Phys. Lett.* **114**, 211101 (2019).
28. Y. Winetraub, E. D. SoRelle, O. Liba, A. de la Zerda, Quantitative contrast-enhanced optical coherence tomography. *Appl. Phys. Lett.* **108**, 023702 (2016).
29. M. Yu Kirillin, A. V. Priezzhev, R. Myllylä, Role of multiple scattering in formation of OCT skin images. *Quantum Electron.* **38**, 570–575 (2008).
30. A. Dubois, O. Levecq, H. Azimani, D. Siret, A. Barut, M. Suppa, V. Del Marmol, J. Malvehy, E. Cinotti, P. Rubegni, J.-L. Perrot, Line-field confocal optical coherence tomography for high-resolution noninvasive imaging of skin tumors. *J. Biomed. Opt.* **23**, 1–9 (2018).

31. J. Breugnot, P. Rouaud-Tinguely, M. Pedrazzani, M. Cazalas, A. Davis, S. Bordes, A. Dubois, B. Closs, Comparison between LC-OCT imaging and histology to quantify the superficial dermis thickness. *Skin Res. Technol.* **26**, 398–404 (2020).
32. B. Yin, C. Hyun, J. A. Gardecki, G. J. Tearney, Extended depth of focus for coherence-based cellular imaging. *Optica* **4**, 959–965 (2017).
33. D. Mandache, E. Dalimier, J. R. Durkin, C. Boceara, J.-C. Olivo-Marin, V. Meas-Yedid, Basal cell carcinoma detection in full field OCT images using convolutional neural networks, in *2018 IEEE 15th International Symposium on Biomedical Imaging (ISBI)* (IEEE, 2018), pp. 784–787.
34. M. Macenko, M. Niethammer, J. S. Marron, D. Borland, J. T. Woosley, Xiaojun Guan, C. Schmitt, N. E. Thomas, "A method for normalizing histology slides for quantitative analysis," in *2009 IEEE International Symposium on Biomedical Imaging: From Nano to Macro* (IEEE, 2009), pp. 1107–1110.
35. P. Isola, J.-Y. Zhu, T. Zhou, A. A. Efros, Image-to-image translation with conditional adversarial networks. arXiv:1611.07004 [cs.CV] (21 November 2016).
36. T.-C. Wang, M.-Y. Liu, J.-Y. Zhu, A. Tao, J. Kautz, B. Catanzaro, High-resolution image synthesis and semantic manipulation with conditional GANs. arXiv:1711.11585 [cs.CV] (22 November 2017).
37. J.-Y. Zhu, T. Park, P. Isola, A. A. Efros, Unpaired image-to-image translation using cycle-consistent adversarial networks. arXiv:1703.10593 [cs.CV] (24 November 2017).
38. J. P. Cohen, M. Luck, S. Honari, Distribution matching losses can hallucinate features in medical image translation. arXiv:1805.08841 [cs.CV] (3 October2018).

Acknowledgments: We would like to thank A. Ngoc Diep, M. Resnick, B. Garimella, B. Ronain Smith, E. SoRelle, K. Lee, L. Nguyen, M. Rose Hays, M. Prigozhin, P. Si, T. Winetraub, V. Hua, P. Chu, Z. Lautman, J. Strommer, and G. P. Collins for the contributions. **Funding:** This work was supported by the U.S. Air Force grant FA9550-15-1-0007; National Institutes of Health grants NIH DP50D012179, DP5OD031858, and K23CA211793; National Science Foundation grant NSF 1438340; Damon Runyon Cancer Research Foundation grant DFS# 06-13; the Claire Giannini Fund; the Susan G. Komen Breast Cancer Foundation grant SAB15-00003; the Mary Kay Foundation grant 017-14; the Skippy Frank Foundation; the Donald E. and Delia B. Baxter Foundation; the Center for Cancer Nanotechnology Excellence and Translation seed grant NIH-NCI U54CA151459; the Stanford Bio-X Interdisciplinary Initiative seed grants IIP6-43 and IIP11-9; the Stanford Bowes Bio-X Graduate Fellowship Stanford Biophysics Program training grant T32GM-08294; the Stanford Office of Technology Licensing grant 323728; the NSF graduate fellowship; the Chan Zuckerberg Biohub investigator grant; the Pew Charitable Trusts and The Alexander and Margaret Stewart Trust; the Pew-Stewart Scholar for Cancer Research grant; and D. G. "Mitch" Mitchell Clinical Investigator grant CI-104-19. **Author contributions:** Y.W. conceived of the presented idea. Y.W. developed the theory behind the registration algorithm. Y.W., E.Y., and I.T. performed the computation of the registration algorithm. E.Y. and Y.W. performed numerical simulations for the registration. E.Y., I.T., and Y.W. developed the machine learning theory and performed the computation. Software supporting this project was developed by Y.W., E.Y., I.T., and E.B. I.T., E.Y., Y.W., C.Y., E.V.V., J.Z., and E.B. developed and performed instrument calibrations. Y.W., E.Y., I.T., C.Y., S.S., E.V.V., and M.H. developed the gel embedding protocol used in the registration step. S.A., W.C., H.D., and E.V.V. collected samples from patients and coordinated secure transportation to our imaging facility. C.Y., S.S., M.M., J.Y., M.H., and A.V.V. imaged and processed samples to generate the dataset used in this paper. A.d.l.Z., K.Y.S., S.A., S.C., and K.E.R. contributed to the overall design and direction of the research. The manuscript was written through contributions of all authors. All authors have given approval to the final version of the manuscript. **Competing interests:** Y.W., E.Y., I.T., C.Y., and A.d.l.Z. are inventors on patent application 62/832093 submitted by Stanford University that covers "High resolution alignment of optical coherence tomography volumes with histo-pathology section". The authors declare that they have no other competing interests. **Data and materials availability:** All data needed to evaluate the conclusions in the paper are present in the paper and/or the Supplementary Materials. Source code for this project is available in GitHub (https://github.com/YoWinetraub/OCT2Hist) and in Zenodo (https://zenodo.org/records/10059597).

Submitted 17 May 2023
Accepted 7 March 2024
Published 10 April 2024
10.1126/sciadv.adi5794
